# The impact of mild renal dysfunction on isolated cardiopulmonary coronary artery bypass grafting: a retrospective propensity score matching analysis

**DOI:** 10.1186/s13019-019-0998-4

**Published:** 2019-11-07

**Authors:** Xian Wang, Yifan Zhu, Wen Chen, Liangpeng Li, Xin Chen, Rui Wang

**Affiliations:** 1 Department of Cardiovascular Surgery, Nanjing First Hospital, Nanjing Medical University, Nanjing, 68 Changle Rd, Nanjing, 210006 People’s Republic of China; 2 Department of Laboratory Medicine, Nanjing Drum Tower Hospital, Nanjing University Medical School, Nanjing, 321 Zhongshan Rd, Nanjing, 210008 People’s Republic of China

**Keywords:** Coronary artery bypass grafting, Cardiopulmonary, Glomerular filtration rate, Mild renal dysfunction, In-hospital outcomes, Long-term survival, Dialysis

## Abstract

**Background:**

Mild preoperative renal dysfunction (RD) is not rare in patients receiving isolated cardiopulmonary coronary artery bypass grafting (CCABG). However, there are not too many studies about the impact of mild preoperative RD on in-hospital and follow-up outcomes after isolated CCABG. This single-centre, retrospective propensity score matching study designed to study the impact of mild preoperative RD on in-hospital and long-term outcomes after first isolated CCABG.

**Methods:**

After propensity score matching, 1144 patients with preoperative estimated glomerular filtration rate (eGFR) of more than 60 ml/min/1.73 m^2^ receiving first isolated CCABG surgery from January 2012 to December 2015 entered the study, who were divided into 2 groups: A group (eGFR ≥90 ml/min/1.73 m2, *n* = 572) and B group (eGFR of 60–89 ml/min/1.73 m2, n = 572). The in-hospital and long-term outcomes were recorded and analyzed. The mean follow-up time was 54.4 ± 10.7 months. Acute kidney injury (AKI) was defined and classified according to the Acute Kidney Injury Network (AKIN) criteria.

**Results:**

The 2 propensity score-matched groups had similar baseline and procedure except the baseline eGFR. There were 8 patients died in A group (mortality is 1.4%) and 14 died in B group (mortality is 2.5%) during the in hospital and 30-day postoperatively(χ^2^ = 1.159, *p* = 0.282). There were totally 38 patients lost to follow-up, 18 in group A and 20 in group B. 21 patients died in group A and 37 died in group B during the follow-up, and long-term survival in group A was higher than in group B (96.2% vs 93.1%, χ^2^ = 4.336, *p* = 0.037). Comparing with group A, group B was associated with an increased rates and severity of AKI postoperatively (total AKI: 62 vs 144. AKIN stageI: 54 vs 113; AKIN stageII: 6 vs 22; AKIN stageIII: 2 vs 9, p<0.0001). During follow-up, group B also had a higher rate of new onset of dialysis (0 vs 6, χ^2^ = 4.432, *p* = 0.039). Multivariable logistic regression showed that comparing with A group, the HR for long-term mortality and new onset of dialysis in B group was 1.67 and 1.52 respectively (95%CI 1.09–2.90, *p* = 0.035; 95%CI 1.14–2.49, *p* = 0.027).

**Conclusions:**

Comparing with normal preoperative renal function, patients with mild preoperative RD had a similar in-hosptial mortality, but with an increased in-hosptial rates and severity of AKI, and with a decreased long-term survival and increased long-term new onset of dialysis.

## Background

Nowadays, coronary artery bypass grafting surgery (CABG) is recognized as one of the most effective procedures for the treatment of coronary artery atherosclerosis disease. Patients with renal dysfunction (RD) are known to be at higher risk for mortality after CABG [1]. However, there is limited information on effects of different degrees of RD based on estimated glomerular filtration rate (eGFR), especially the studies focused on the mild preoperative RD are relatively rare [2].

The majority of previous studies usually utilized serum creatinine as the indicator of the severity of RD, but compared with creatinine levels, eGFR has been considered as the most reliable indicator of renal function according to the National Kidney Foundation guidelines [3, 4]. RD caused by cardiopulmonary bypass can be attributed to inflammatory response, nonpulsatile flow, hemodilution, renal hypoperfusion, low cardiac output syndrome, atheroembolism, increased levels of circulating catecholamines, and free hemoglobin [5, 6]. However, studies conducted previously in this field have provided conflicting outcomes to support this hypothesis [7, 8].

In order to avoid the bias of cardiopulmonary bypass (CPB), we only enrolled patients with preoperative eGFR of more than 60 ml/ min/1.73 m^2^ undergoing first isolated cardiopulmonary coronary artery bypass grafting (CCABG) surgery. The purpose of this study was to understand the impact of mild preoperative RD compared with normal renal function on in-hospital and long-term outcomes in a single centre, retrospective propensity score matching study.

## Methods

### Definition of renal function

The glomerular filtration rate (GFR) was calculated by the abbreviated Modification of Diet in Renal Disease equation:186 × (serum creatinine/88.4)^-1.154^ × (age)^-0.203^ × (0.742 if female). Kidney function before CABG was graded from I to V according to the GFR as proposed by the Kidney Disease Outcome Quality Initiative [9]. Kidney function is defined as normal with a GFR more than 89 ml/min/1.73 m^2^ (stage I), minimally reduced with a GFR between 60 and 89 (stage II), moderately reduced with a GFR between 30 and 59 (stage III), severely reduced with a GFR between 15 and 29 (stage IV), and end-stage kidney failure with a GFR below 15 or renal replacement (stage V). This study only enrolled patients with mild preoperative RD (GFR between 60 and 89 ml/min/1.73 m^2^) and patients with normal preoperative renal function (GFR more than 90 ml/min/1.73 m^2^).

### Study population

A standard set of perioperative data was collected prospectively for all patients undergoing first isolated CCABG with preoperative eGFR of more than 60 ml/min/1.73 m^2^ at Nanjing First Hospital between January 2010 to December 2015. Clinical data were retrospectively collected from medical records and the data-base of our department of cardiac surgery.

All CCABG surgeries were performed by the one surgeon. Patients undergoing a concomitant cardiac surgical procedure, reoperation, urgent or emergent operations, or with incomplete informations were excluded. CCABG was performed via median sternotomy using a membrane oxygenator equipped with an arterial filter, cold blood antegrade cardioplegia under moderate systemic hypothermia (30 to 34 °C). The perfusion pressure during CPB was maintained within 60–70 mmHg.

Totally there were 1592 cases up to the standard, normal preoperative renal function was found in 1020 patients (64.1%, A group) and mild preoperative RD in 572 patients (35.9%, B group).

To control the selection bias in the comparison among A group and B group, a propensity score (PS) analysis was performed. One PS was calculated for each patient by means of logistic-regression analysis using 18 preoperative and surgical variables including: age, gender, body mass index (BMI), smoking, hypertension, diabetes mellitus (DM), hyperlipemia, chronic obstructive pulmonary disease (COPD), prior cerebro-vascular accident, history of cerebral and myocardial infarction (MI), history of percutaneous coronary intervention (PCI), left ventricular ejection fraction (LVEF), number of vessel disease, Euro-SCOREII, number of distal anastomosis, the application of left internal mammary artery (LIMA) and radial artery and CPB time. Every patient with mild preoperative RD was matched with a patient with normal renal function with the closest PS (within0.030). Finally, 572 pairs were successfully built in a 1:1 manner through matching PS(A group, *n* = 572; B group, *n* = 572).

### Statistical analysis

Data are represented as the mean ± standard deviation (SD) unless otherwise indicated. Categorical variables are represented as frequency distributions and single percentages. Normally distributed continuous variables were compared using a Student t-test, non-normally distributed continuous variables using the Mann-Whitney U test, and categorical variables were compared by χ2 test. Potential risk factors were calculated by Cox regression analysis. Potential independent predictors of outcome were identified by univariate Cox regression analysis. All statistical tests were two-sided. A *p*-value of less than 0.05 was considered significant. All statistical analysis were done with IBM SPSS Statistics 20.0 or STATA Data analysis and statistical software.

## Results

### Matching of patients

Matching data from A group(*n* = 572) and B group (*n* = 572) were analyzed. There were no significant differences with regard to age, gender, BMI, smoking, hypertension, DM, hyperlipedemia, COPD, history of cerebral infarction, history of MI, history of PCI, LVEF, number of vessel disease, Euro-SCOREII, number of distal anastomosis, the percentage of arterial grafts and CPB time (Table 1). Significant differences were found on the baseline eGFR, which was higher in A group than B group (101.3 ± 11.4 vs 75.6 ± 9.7, *p* <  0.0001).

**Table 1:** Baseline and procedural characteristics after matching

variable	A group	B group	*p* value
	(*n* = 572) No. (%)	(*n* = 572) No. (%)	
Female gender	97 (17.0)	104 (18.2)	0.641
Age, y	63.2 ± 7.3	63.6 ± 8.1	0.38
Body mass index, kg/m2	26.4 ± 4.5	26.1 ± 4.6	0.265
DM			
Insulin-dependent	89 (15.6)	95 (16.6)	0.687
Non-Insulin-dependent	63 (11.0)	71 (12.4)	0.52
Previous			
Cerebal infarction	28 (4.9)	23 (4.0)	0.567
Myocardial infarction	85 (14.9)	91 (15.9)	0.682
PCI	79 (13.8)	83 (14.5)	0.799
Hypertension	286 (50.0)	317 (55.4)	0.076
COPD	38 (6.6)	44 (8.5)	0.567
Hyperlipemia	143 (0.25)	132 (23.1)	0.489
LVEF			0.388
> 0.50	479 (83.7)	468 (81.8)	
0.30–0.50	87 (15.2)	97 (17.0)	
< 0.30	6 (1.1)	7 (1.2)	
Extent of CAD			0.795
1 vessel	14 (2.5)	13 (2.3)	
2 vessel	69 (12.1)	67 (11.7)	
3 vessel	489 (85.4)	492 (86.0)	
LM	165 (28.8)	151 (26.4)	0.39
eGFR (ml/min/1.73 m2)	101.3 ± 11.4	75.6 ± 9.7	< 0.0001
EuroScoreII	1.9 ± 0.9	2.0 ± 1.2	0.111
Distal anastomosis	3.1 ± 0.6	3.1 ± 0.4	1
LIMA	544 (95.1)	531 (92.8)	0.136
Radial Artery	68 (11.9)	89 (15.6)	0.086
CPB time (min)	73.5 ± 20.2	71.3 ± 23.8	0.092

*DM* diabetes mellitus, *PCI* percutaneous coronary intervention, *COPD* chronic obstructive pulmonary disease, *LVEF* left ventricular ejection fraction, *CAD* coronary artery disease, *eGFR* estimated glomerular filtration rate, *LIMA* left internal mammary artery, *CPB* cardiopulmonary bypass

### Postoperative results

**Fig. 1:**
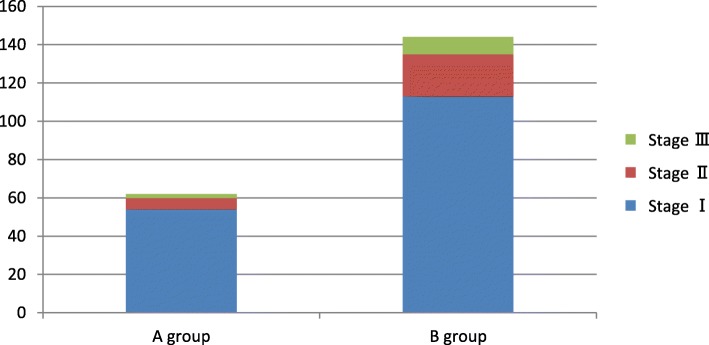
Acute kidney injury (AKI), as defined and classified according to the criteria proposed by the Acute Kidney Injury Network (AKIN), is shown stratified according to kidney function at baseline (blue = AKI 1, red = AKI 2, green = AKI 3; y-axis = number of patients)

A total of 22 patients died after the operation (8 in A group and 14 in B group, *p* = 0.282). In-hospital mortality and morbidity are represented in Table 2. There were no significant differences with regard to in-hospital mortality, myocardial infarction, atrial fibrillation, stroke, respiratory failure, pneumonia, redo for bleeding, red blood cell (RBC) transfusion, deep sternal wound infection (DSWI), low cardiac output syndrome, intra-aortic balloon pump (IABP) support. Comparing with group A, group B was associated with an increased rates and severity of AKI (total AKI: 62 vs 144. AKIN stageI: 54 vs 113; AKIN stageII: 6 vs 22; AKIN stageIII: 2 vs 9, *p*<0.0001.) (Fig. 1).

**Table 2:** Postoperative outcomes in the matched cohort

variable	A group	B group	*p* value
	(*n* = 572) No. (%)	(*n* = 572) No. (%)	
Mortality	8 (1.4)	14 (2.4)	0.282
MI	17 (3.0)	17 (3.0)	1
Stroke	6 (1.0)	8 (1.4)	0.788
Respiratory failure	16 (2.8)	22 (3.8)	0.409
Pneumonia	23 (4.0)	29 (5.1)	0.478
AKI	62	144	<0.0001
Stage I	54 (9.6)	113 (20.3)	
Stage II	6 (1.1)	22 (3.9)	
Stage III	2 (0.4)	9 (1.6)	
Redo for bleeding	9 (1.6)	12 (2.1)	0.66
RBC transfusion	201 (35.1)	219 (38.3)	0.622
DSWI	11 (1.9)	17 (3.0)	0.339
Low cardiac output syndrome	44 (7.7)	54 (9.4)	0.34
IABP application	33 (5.8)	46 (8.0)	0.162
Lost to follow-up	18 (3.2)	20 (3.7)	0.869
Long-term mortality	21 (3.8)	37 (6.9)	0.037
CRF requiring dialysis	0	6 (1.1)	0.039

*MI* myocardial infarction, *AKI* acute kidney injury, *RBC* red blood cell, *DSWI* deep sternal wound infection, *IABP* intra-aortic balloon pump, *CRF* chronic renal failure

### Long-term outcomes

**Fig. 2:**
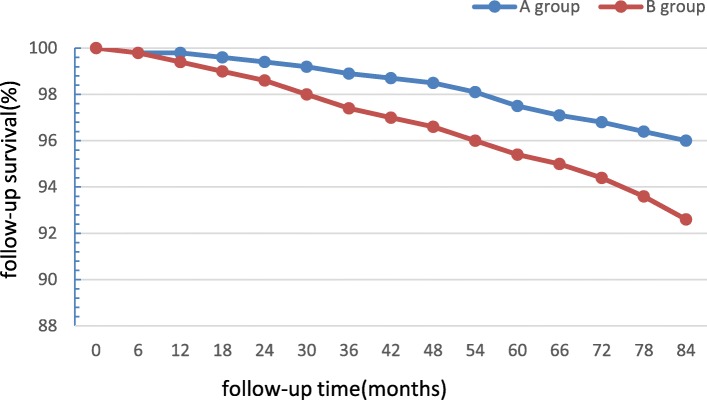
Actuarial curves of long-term follow-up survival after CCABG

There were 58 patients died during the follow-up (21 patients in A group and 37 in B group), and 38 patients were lost to follow-up totally (18 patients in A group and 20 in B group, Table 2). The mean follow-up time was 54.4 ± 10.7 months.The top three causes of death were infection, heart failure, and myocardial infarction. Patients in group A were associated with a higher long-term survival (Fig. 2), and a lower new onset of dialysis rate than patients in group B (long-term mortality, 21 vs 37, long-term survival, 525 vs 501, χ^2^ = 4.336, *p* = 0.037; dialysis, 0 vs 6, χ^2^ = 4.432, *p* = 0.039). Multivariable logistic regression showed that mild preoperative RD grouping was a significant variable related to the long-term survival rate and new onset of dialysis, the HR for long-term mortality and new onset of dialysis in patients with mild preoperative RD was 1.67 and 1.52 respectively compared with normal preoperative renal function (95%CI 1.09–2.90, *p* = 0.035; 95%CI 1.14–2.49, *p* = 0.027). (As shown in Table 3).

**Table 3:** Predictors of long-term mortality/new onset of dialysis in the matched cohorts

Variable	HR	95% CI	*p* value
Grouping (mild group vs. normal group)	1.67/1.52	1.09–2.90/1.14–2.49	0.035/0.027
Age (per y)	1.21/1.19	1.03–1.63/1.17–1.89	<0.0001/0.004
Gender (Female vs. male)	1.29/1.13	1.08–1.84/1.03–2.01	0.037/0.19
High blood pressure	1.47/1.05	1.12–1.97/1.05–1.88	0.008/0.27
DM	1.59/1.33	1.08–1.96/1.01–1.76	0.012/0.035
LVEF<0.50	1.68/2.24	1.04–2.21/1.03–2.25	0.023/0.006
IABP application	2.22/3.17	1.11–2.87/1.05–2.10	<0.0001/0.002

*HR* hazard ratio, *CI* confidence interval, *DM* diabetes mellitus, *LVEF* left ventricular ejection fraction, *IABP* intra-aortic balloon pump

## Discussion

The principal findings of this single-centre, retrospective propensity score matching study can be summarized as follows. Comparing with normal preoperative renal function, mild preoperative RD: (1) did not influence the in-hosptial mortality and most of the postoperative in-hosptial morbidities. (2) increased postoperative in-hosptial rates and severity of AKI. (3) decreased long-term survival and increased long-term new onset of dialysis.

Propensity matched pairs analysis provided the opportunity to rule out confusion by providing balanced baseline and procedural characteristics except the baseline eGFR. In this study, patients with mild preoperative RD or with normal preoperative renal function had similar rates of in-hospital surgical mortality. The univariate factor analysis manifested that the 2 propensity score-matched groups had similar in-hospital outcomes, including surgical mortality, myocardial infarction, stroke, respiratory failure, pneumonia, redo for bleeding, RBC transfusion, DSWI, low cardiac output syndrome, IABP application, except the rates and severity of AKI. The same result was obtained by Weitie Wang et al., they reported older patients with mild preoperative RD had a higher mortality rate than normal patients in long-term survival, whereas no evidence of worse in-hospital mortality rate was found [10]. Jyrala et al. who analyzed a cohort of 885 patients with or without mild preoperative RD received on-pump cardiac surgery, with respect to short- and long-term outcomes [11]. They found mild preoperative RD was a marker for patients with increased cardiac risk factors and the risk for poor outcomes. Their conclusion was in line with our study about postoperative late survival but was different from in-hospital mortality. Reasons of this difference lay in the study population, because our cohort was primary isolated CCABG, and we chose the eGFR as the indicator for the evaluation of renal function.

CPB still contributes to RD due to multiple perturbations in renal physiology and function as mentioned before. We speculated the reasons why there was no difference of mortality between the 2 groups were as follows: (1) average CPB time of primary isolated CABG was relatively short, most of patients with mild RD had enough functional reserve and had the ability to withstand acute insults. (2) group B had an increased postoperative in-hosptial rates and severity of AKI, but AKIN stage I accounted for most cases. (3) relative high perfusion pressure was kept to guarantee the renal perfusion [12]. (4) There are no active treatments for AKI, and therefore, perioperative preventative strategies seem particularly promising. Keep adequate hydration and avoid the use of diuretics, except for specified medical indications; minimize the use of medications with adverse effects on renal function, such as contrast agents, α-adrenergic agents, colloids and exogenous blood products; keep an optimal hemodynamic status and correct the acid-base or metabolic imbalance.

It is reported that age, DM, preoperative RD, blood transfusion, low cardiac output syndrome, low LVEF are independent risk factors of AKI [13, 14]. There was no significant difference of these independent risk factors except the baseline eGFR between the 2 propensity score-matched groups. In this study, compared with patients with normal preoperative renal function, patients with mild preoperative RD were associated with increased postoperative in-hosptial rates and severity of AKI. The severity of AKI was defined by the AKIN [15], AKIN stage I accounted for most patients in both groups in this study. AKI defines as a clinical syndrome characterized by a sudden decline of the excretory kidney function, with accumulation of urea and creatinine and decreased urinary output [16, 17]. Depending on the specific definition and methodological concerns, AKI occurs in up to one third of patients undergoing CABG, and approximately 2% require temporary dialysis [18]. The AKIN criteria may be quite sensitive for detecting subtle changes in kidney function, especially the creatinine increase of 0.3 mg/dL within 2 days. Some previous studies demonstrated that development of AKI was associated with high short-term and long-term morbidity and mortality [19]. The demonstration was consistent with our study about the long-term mortality but was different from postoperative short-term mortality. Reason of this difference was supposed that we only recruited first isolated CCABG with normal or mild RD, and AKIN stage I accounted for most patients postoperatively, and higher incidence of complete revascularization achieved by CCABG may also be a possible explanation. Another hypothesis was that the increased hemodynamic instability that occured with off-pump CABG negate the benefits of avoiding CPB [20].

As expected, the follow-up demonstrated that patients with mild preoperative RD showed a trend to lower long-term survival compared with normal preoperative renal function patients (96.2% vs 93.1%, *p* = 0.037). Cox regression manifested that grouping (mild vs. normal preoperative renal function) was a significant variable related to the long-term survival, and the HR was 1.67 (95%CI 1.09–2.90, *p* = 0.035). Furthermore, in our study patients with mild RD were associated with a higher rate of new onset of dialysis compared with normal renal function patients during the follow-up (0 vs 6, χ^2^ = 4.432, *p* = 0.039). Cox regression also showed the HR for new onset of dialysis was 1.52(mild vs normal preoperative renal function, 95%CI 1.14–2.49, *p* = 0.027). The exact causes of these differences are unknown and further research is needed. Preexisting kidney disease has been repeatedly identified as a strong predictor of AKI after cardiac operations [21], moreover, AKI has been associated with progression to chronic kidney disease (CKD) and dialysis in many reports [22]. CKD and dialysis might exert negative efferts on the long-term survival inevitably. Günday and colleagues [23] conducted a study focused on the CABG patients with normal renal function or mild RD, they concluded that mild preoperative RD reduced coronary flow reserve after CABG surgery due to deterioration of the micro-vascular bed. Unfortunately, there are no pharmacologic agents known to reduce the risk of AKI or treat established AKI [24]. Therefore, CABG patients with mild RD need to strengthen the follow-up of nephropathy, more strictly management of the risk factors of coronary artery disease postoperatively.

### Limitations

Firstly, a retrospective, non-randomized single-centre analysis over a long period of time and with different surgeon’s procedures on patients undergoing CABG is subjected to the effects of selection bias. Although propensity score matching is implemented, a prospective, multi-centre study involving larger sample size is needed. Secondly, the GFR was derived by using the MDRD formula, which was not designed for determining GFR. Finally, the initial RIFLE (risk of RD; injury to the kidney; failure of kidney function; loss of kidney function; and end-stage kidney disease) criteria do not include the creatinine increase within 48 h for defining the mildest form of AKI, whereas the RIFLE categories injury and loss are similar to AKIN stage 2 and 3, respectively [25]. AKI defined according to the RIFLE criteria may therefore avoid an iatrogenic hemodilution but may miss more subtle changes of kidney function [19, 25].

## Conclusions

In summary, this analysis revealed that mild preoperative RD compared with normal preoperative renal function did not increase the in-hosptial mortality, but increased in-hosptial rates and severity of AKI, decreased long-term survival and increased long-term new onset of dialysis.

## Data Availability

All data and material are available by contacting wr1582@163.com

## Abbreviations

AKI: Acute kidney injury
AKIN: Acute kidney injury network
BMI: Body mass index
CABG: Coronary artery bypass grafting surgery
CCABG: Cardiopulmonary coronary artery bypass grafting
CKD: Chronic kidney disease.
COPD: Chronic obstructive pulmonary disease
CPB: Cardiopulmonary bypass
DM: Diabetes mellitus
DSWI: Deep sternal wound infection
eGFR: estimated glomerular filtration rate;
GFR: Glomerular filtration rate
IABP: Intra-aortic balloon pump
LIMA: Left internal mammary artery
LVEF: Left ventricular ejection fraction
MI: Myocardial infarction
PCI: Percutaneous coronary intervention
PS: Propensity score
RBC: Red blood cell

## References

[1] García Fuster R, Paredes F, García Peláez A (2013). Impact of increasing degrees of renal impairment on outcomes of coronary artery bypass grafting: the off-pump advantage. Eur J Cardiothorac Surg.

[2] Litmathe J, Kurt M, Feindt P (2009). The impact of pre- and postoperative renal dysfunction on outcome of patients undergoing coronary artery bypass grafting (CABG). Thorac Cardiovasc Surg.

[3] Lunney M, Alrukhaimi M, Ashuntantang GE (2018). Guidelines, policies, and barriers to kidney care: findings from a global survey. Kidney Int Suppl (2011).

[4] Levey AS, Coresh J, Balk E (2003). National Kidney Foundation practice guidelines for chronic kidney disease: evaluation, classification and stratification. Ann Intern Med.

[5] Lannemyr L, Bragadottir G, Krumbholz V (2017). Effects of Cardiopulmonary Bypass on Renal Perfusion, Filtration, and Oxygenation in Patients Undergoing Cardiac Surgery. Anesthesiology..

[6] Pickering JW, James MT, Palmer SC (2015). Acute kidney injury and prognosis after cardiopulmonary bypass: a meta-analysis of cohort studies. Am J Kidney Dis.

[7] Mao H, Katz N, Ariyanon W (2013). Cardiac surgery-associated acute kidney injury. Cardiorenal Med.

[8] Wang Y, Bellomo R (2017). Cardiac surgery-associated acute kidney injury: risk factors, pathophysiology and treatment. Nat Rev Nephrol.

[9] K/DOQI Clinical Practice Guidelines for Chronic Kidney Disease: evaluation, classification, and stratification (2002). Part 4: definition and classification of stages of chronic kidney disease. Am J Kidney Dis.

[10] Wang W, Wang Y, Xu R (2018). Outcomes following coronary artery bypass graft surgery in patients with mild preoperative renal insufciency. Braz J Cardiovasc Surg.

[11] Jyrala A, Weiss RE, Jeffries RA (2010). Effect of mild renal dysfunction (s-crea1.2-2.2 mg/dl) on presentation characteristics and short- and long-term outcomes of on-pump cardiac surgery patients. Interact Cardiovasc Thorac Surg.

[12] Sgouralis I, Evans RG, Gardiner BS (2015). Renal hemodynamics, function, and oxygenation during cardiac surgery performed on cardiopulmonary bypass: a modeling study. Physiol Rep.

[13] Landoni G, Bove T, Crivellari M (2007). Acute renal failure after isolated CABG surgery: six years of experience. Minerva Anestesiol.

[14] Helgadottir S, Sigurdsson MI, Palsson R (2016). Renal recovery and long-term survival following acute kidney injury after coronary artery surgery: a nationwide study. Acta Anaesthesiol Scand.

[15] Kara I, Yildirim F, Kayacan E (2017). Importance of RIFLE (Risk, Injury, Failure, Loss, and End-Stage Renal Failure) and AKIN (Acute Kidney Injury Network) in Hemodialysis Initiation and Intensive Care Unit Mortality. Iran J Med Sci.

[16] Bellomo R, Kellum JA, Ronco C (2012). Acute kidney injury. Lancet..

[17] Ostermann M, Liu K (2017). Pathophysiology of AKI. Best Pract Res Clin Anaesthesiol.

[18] Rosner MH, Okusa MD (2006). Acute kidney injury associated with cardiac surgery. Clin J Am Soc Nephrol.

[19] Reents W, Hilker M, Börgermann J (2014). Acute kidney injury after on-pump or off-pump coronary artery bypass grafting in elderly patients. Ann Thorac Surg.

[20] Schwann NM, Horrow JC, Strong MD (2004). Does off-pump coronary artery bypass reduce the incidence of clinically evident renal dysfunction after multivessel myocardial revascularization?. Anesth Analg.

[21] Karkouti K, Wijeysundera DN, Yau TM (2009). Acute kidney injury after cardiac surgery: focus on modifiable risk factors. Circulation.

[22] Chawla LS, Eggers PW, Star RA (2014). Acute kidney injury and chronic kidney disease as interconnected syndromes. N Engl J Med.

[23] Günday M, Çiftçi Ö, Çalışkan M (2014). Does mild renal failure affect coronary flow reserve after coronary artery bypass graft surgery?. Heart Surg Forum.

[24] Thiele RH, Isbell JM, Rosner MH (2015). AKI associated with cardiac surgery. Clin J Am Soc Nephrol.

[25] Bellomo R, Ronco C, Kellum JA, Metha RL, Palevsky P, the ADQI workgroup (2004). Acute renal failure–definition, outcome measures, animal models, fluid therapy and information technology needs: the second international consensus conference of the acute Dialysis quality initiative (ADQI) group. Crit Care.

